# Supplementary material for: The origin of widespread species in a poor dispersing lineage (diving beetle genus Deronectes)
Source: PeerJ. 2016 Sep 27;4:e2514. doi: 10.7717/peerj.2514 (PMC5045878; doi:10.7717/peerj.2514)

The origin of widespread species in a poor dispersing lineage (diving beetle genus *Deronectes*)

David García-Vázquez and Ignacio Ribera

Institute of Evolutionary Biology (CSIC-Universitat Pompeu Fabra), Passeig Maritim de la Barceloneta 37, 08003 Barcelona, Spain

ELECTRONIC SUPLEMENTAL INFORMATION

Table S1. List of the specimens included in the phylogeography, with specimen voucher, locality, collector, Genbank accession numbers and specimens tested for infection of *Wolbachia*. In grey, species in other groups of *Deronectes* used as outgroups. In bold, sequences newly obtained for this study.

The origin of widespread species in a generally poor dispersing lineage (diving beetle genus *Deronectes*)

García-Vázquez, D. & Ribera, I.

Appendix S1: Additional materials.

Table S1.List of the specimens included in the phylogeography, with specimen voucher, locality, collector, Genbank accession numbers and specimens tested for infection of Wolbachia. In grey, species in other groups of *Deronectes* used as outgroups. In bold, sequences newly obtained for this study.

								Accesion numbers						Wolbachia	Wolbachia
No	Species	Voucher	Country (Island)	Locality	Locality cod	Coordinates	Collector	**COI-5'**	**COI-3'**	**16SrRNA+tRNA-Leu+NAD1**	**H3**	**Wingless**	**Wsp (wolbachia)**	**Infected**	**Supergroup**
1	*D. angelinii*	IBE-RA234	Italy	Marche, Ascoli-Piceno province, Quintodecimo	ITACN2	ca. 42°45'48"N 13°23'13"E	M. Toledo	LN995087	LN995061	LN995162	LN995127	**LT602418**	**LT602610**	YES	B
2	*D. angelinii*	NHM-IR301	Italy	Lombardia, Pavia province, San Ponzo	ITAN4	ca. 44°50'23"N 9° 6'23"E	I. Ribera & A. Cieslak		**LT601818**	**LT602509**				-	
3	*D. angusi*	IBE-DV67	Spain	Galicia, Lugo province, Viveiro	ESPNNO17	ca. 43°37'17"N 7°38'11"W	C.Benneti	**LT602087**	**LT601819**	**LT602510**	**LT602335**	**LT602419**		NO	
4	*D. angusi*	IBE-DV68	Spain	Galicia, Lugo province, Viveiro	ESPNNO17	ca. 43°37'17"N 7°38'11"W	C.Benneti	**LT602088**	**LT601820**					-	
5	*D. angusi*	IBE-RA442	Spain	Galicia, Lugo province, Quintá	ESPNNO6	ca. 42°58'18"N 7°13'14"W	I. Ribera	**LT602089**	**LT601821**					-	
6	*D. angusi*	IBE-RA443	Spain	Galicia, Lugo province, Quintá	ESPNNO6	ca. 42°58'18"N 7°13'14"W	I. Ribera	LN995088	LN995063	LN995163	**LT602336**	**LT602420**		-	
7	*D. angusi*	MNHN-AI1273	Spain	Castilla y León, Burgos province, Pineda de la Sierra	ESPNNO2	ca. 42°13'40"N 3°18'15"W	D.T. Bilton	**LT602090**	**LT601822**		**LT602337**			-	
8	*D. angusi*	NHM-IR44	Spain	Galicia, Lugo province, Quintá	ESPNNO6	ca. 42°58'18"N 7°13'14"W	I. Ribera		AF309310	AF309253	EF670135			-	
9	*D. angusi*	NHM-IR253	Spain	Castilla y León, Burgos province, Pineda de la Sierra	ESPNNO4	ca. 42°13'40"N 3°18'15"W	I. Ribera		LN995062	AF309253				-	
10	*D. aubei aubei*	IBE-DV60	Italy	Lombardia, Brescia province, Val Palot	ITAN7	ca. 45°47'22"N 10°10'38"E	M. Toledo	**LT602091**	**LT601823**	**LT602511**	**LT602338**	**LT602421**		YES	undetermined
11	*D. aubei aubei*	IBE-DV61	Italy	Lombardia, Brescia province, Val Palot	ITAN7	ca. 45°47'22"N 10°10'38"E	M. Toledo	**LT602092**	**LT601824**					-	
12	*D. aubei aubei*	IBE-DV138	Germany	Baden Württemberg, Karlsruhe region, Eyachmühle	ALE3	48°48'30.8"N 8°33'27.1"E	I. Ribera & A. Cieslak	**LT602093**	**LT601825**	**LT602512**	**LT602339**	**LT602422**		YES	undetermined
13	*D. aubei aubei*	IBE-DV139	Germany	Baden Württemberg, Karlsruhe region, Eyachmühle	ALE3	48°48'30.8"N 8°33'27.1"E	I. Ribera & A. Cieslak	**LT602094**	**LT601826**	**LT602513**	**LT602340**			-	
14	*D. aubei aubei*	IBE-DV144	Germany	Baden Württemberg, Karlsruhe region, Buhlbach	ALE4	48°31'58.9"N 8°16'35.4"E	I. Ribera & A. Cieslak	**LT602095**	**LT601827**	**LT602514**	**LT602341**			-	
15	*D. aubei aubei*	IBE-DV145	Germany	Baden Württemberg, Karlsruhe region, Buhlbach	ALE4	48°31'58.9"N 8°16'35.4"E	I. Ribera & A. Cieslak	**LT602096**	**LT601828**	**LT602515**	**LT602342**	**LT602423**	**LT602611**	YES	A
16	*D. aubei aubei*	IBE-DV150	Italy	Lombardia, Brescia province, Val Palot	ITAN7	ca. 45°47'22"N 10°10'38"E	M. Toledo	**LT602097**	**LT601829**	**LT602516**	**LT602343**	**LT602424**		-	
17	*D. aubei aubei*	IBE-DV165	Italy	Lombardia, Brescia province, Val Palot	ITAN7	ca. 45°47'22"N 10°10'38"E	M. Toledo	**LT602098**						-	
18	*D. aubei aubei*	IBE-DV170	Germany	Baden Württemberg, Karlsruhe region, Eyachmühle	ALE3	48°48'30.8"N 8°33'27.1"E	I. Ribera & A. Cieslak	**LT602099**	**LT601830**					-	
19	*D. aubei aubei*	IBE-DV171	Germany	Baden Württemberg, Karlsruhe region, Eyachmühle	ALE3	48°48'30.8"N 8°33'27.1"E	I. Ribera & A. Cieslak	**LT602100**	**LT601831**					-	
20	*D. aubei aubei*	IBE-DV174	Germany	Baden Württemberg, Karlsruhe region, Buhlbach	ALE4	48°31'58.9"N 8°16'35.4"E	I. Ribera & A. Cieslak	**LT602101**	**LT601832**					-	
21	*D. aubei aubei*	IBE-DV185	Germany	Baden Württemberg, Karlsruhe region, Buhlbach	ALE4	48°31'58.9"N 8°16'35.4"E	I. Ribera & A. Cieslak	**LT602102**						-	
22	*D. aubei aubei*	IBE-RA135	Italy	Emilia-Romagna, Modena province, Fanano	ITAN5	ca. 44°10'29"N 10°47'56"E	M. Toledo	LN995089	LN995064	LN995164	LN995128	**LT602425**	**LT602612**	YES	A
23	*D. aubei aubei*	IBE-RA139	Italy	Lombardia, Brescia province, Val Palot	ITAN7	ca. 45°47'22"N 10°10'38"E	M. Toledo	**LT602103**	**LT601833**					-	
24	*D. aubei aubei*	MNCN-AI299	Italy	Lombardia, Brescia province, Val Trompia	ITAN3	45°49'52"N 10°23'58"E	I. Ribera & A. Cieslak	**LT602104**						-	
25	*D. aubei aubei*	MNCN-AI1211	Italy	Lombardia, Brescia province, Val Trompia	ITAN3	45°49'52"N 10°23'58"E	I. Ribera & A. Cieslak	**LT602105**	**LT601834**	**LT602517**	**LT602344**	**LT602426**		-	
26	*D. aubei aubei*	NHM-IR300	France	Provence-Alpes-Cote D´azur, Alpes-Maritimes department, Moulinet	FRAE3	ca. 43°58'34"N 7°24'44" E	I. Ribera & A. Cieslak		AF309326	AF309269	EF670136			-	
27	*D. aubei aubei*	NHM-IR304	Switzerland	Canton of Ticino, Leventina district, Giornico	SUI1	ca. 46°24'20"N 8°52'16"E	I. Ribera & A. Cieslak		**LT601835**	**LT602518**				-	
28	*D. aubei sanfilippoi*	IBE-DV9	Spain	Cantabria, Urdón river	ESPNNO10	43°16'0.76"N 4°37'59.31"W	A. Millán et col.	**LT602106**	**LT601836**	**LT602519**	**LT602345**	**LT602427**		NO	
29	*D. aubei sanfilippoi*	IBE-DV10	Spain	Cantabria, Urdón river	ESPNNO10	43°16'0.76"N 4°37'59.31"W	A. Millán et col.	**LT602107**	**LT601837**					-	
30	*D. aubei sanfilippoi*	IBE-DV11	Spain	Cantabria, Canal del Valle	ESPNNO11	ca. 43°12'54"N 4°39'15"W	A. Millán et col.	**LT602108**	**LT601838**	**LT602520**	**LT602346**	**LT602428**		NO	
31	*D. aubei sanfilippoi*	IBE-DV12	Spain	Cantabria, Canal del Valle	ESPNNO11	ca. 43°12'54"N 4°39'15"W	A. Millán et col.	**LT602109**	**LT601839**					-	
32	*D. aubei sanfilippoi*	IBE-DV13	Spain	Castilla y León, León province, Picos de Europa	ESPNNO12	ca. 43°6'34"N 4°56'40"W	A. Millán et col.	**LT602110**	**LT601840**	**LT602521**	**LT602347**	**LT602429**		NO	
33	*D. aubei sanfilippoi*	IBE-DV14	Spain	Cataluña, Lleida province, San Juan de Torán	ESPPIR14	42°49'30.09"N 0°46'40.19"E	I. Ribera & A. Cieslak	**LT602111**	**LT601841**	**LT602522**	**LT602348**	**LT602430**		YES	undetermined
34	*D. aubei sanfilippoi*	IBE-DV15	Spain	Cataluña, Lleida province, San Juan de Torán	ESPPIR14	42°49'30.09"N 0°46'40.19"E	I. Ribera & A. Cieslak	**LT602112**	**LT601842**					-	
35	*D. aubei sanfilippoi*	IBE-DV16	Spain	Cataluña, Lleida province, San Juan de Torán	ESPPIR14	42°49'30.09"N 0°46'40.19"E	I. Ribera & A. Cieslak	**LT602113**	**LT601843**					-	
36	*D. aubei sanfilippoi*	IBE-DV20	Spain	Cantabria, Vada (Quiviesa river)	ESPNNO14	43°5'22"N 4°40'30"W	L.F. Valladares	**LT602114**	**LT601844**	**LT602523**	**LT602349**	**LT602431**		NO	
37	*D. aubei sanfilippoi*	IBE-DV21	Spain	Cantabria, Vada (Quiviesa river)	ESPNNO14	43°5'22"N 4°40'30"W	L.F. Valladares	**LT602115**	**LT601845**					-	
38	*D. aubei sanfilippoi*	IBE-DV22	Spain	Cantabria, Vada (Quiviesa river)	ESPNNO14	43°5'22"N 4°40'30"W	L.F. Valladares	**LT602116**	**LT601846**					-	
39	*D. aubei sanfilippoi*	IBE-DV23	Spain	Cantabria, Bárago	ESPNNO15	ca. 43° 4'23"N 4°37'06"W	L.F. Valladares	LN995090	LN995065	LN995165	LN995129	**LT602432**		NO	
40	*D. aubei sanfilippoi*	IBE-DV24	Spain	Cantabria, Bárago	ESPNNO15	ca. 43° 4'23"N 4°37'06"W	L.F. Valladares	**LT602117**	**LT601847**					-	
41	*D. aubei sanfilippoi*	IBE-DV25	Spain	Cantabria, Bárago	ESPNNO15	ca. 43° 4'23"N 4°37'06"W	L.F. Valladares	**LT602118**	**LT601848**					-	
42	*D. aubei sanfilippoi*	IBE-DV26	Spain	Cantabria, Vada (Vejo river)	ESPNNO16	43°5'24"N 4°40'33"W	L.F. Valladares	**LT602119**	**LT601849**	**LT602524**				-	
43	*D. aubei sanfilippoi*	IBE-DV27	Spain	Cantabria, Vada (Vejo river)	ESPNNO16	43°5'24"N 4°40'33"W	L.F. Valladares	**LT602120**	**LT601850**					-	
44	*D. aubei sanfilippoi*	IBE-DV28	Spain	Cantabria, Vada (Vejo river)	ESPNNO16	43°5'24"N 4°40'33"W	L.F. Valladares	**LT602121**	**LT601851**					-	
45	*D. aubei sanfilippoi*	IBE-DV97	Spain	Cantabria, Canal del Valle	ESPNNO11	ca. 43°12'54"N 4°39'15"W	A. Millán et col.	**LT602122**	**LT601852**					-	
46	*D. aubei sanfilippoi*	IBE-DV101	Spain	Cataluña, Lleida province, San Juan de Torán	ESPPIR14	42°49'30.09"N 0°46'40.19"E	I. Ribera & A. Cieslak	**LT602123**	**LT601853**	**LT602525**	**LT602350**	**LT602433**		-	
47	*D. aubei sanfilippoi*	IBE-DV102	Spain	Cataluña, Lleida province, San Juan de Torán	ESPPIR14	42°49'30.09"N 0°46'40.19"E	I. Ribera & A. Cieslak	**LT602124**	**LT601854**					-	
48	*D. aubei sanfilippoi*	IBE-DV103	Spain	Cantabria, Vada (Quiviesa river)	ESPNNO14	43°5'22"N 4°40'30"W	L.F. Valladares	**LT602125**	**LT601855**					-	
49	*D. aubei sanfilippoi*	IBE-DV104	Spain	Cantabria, Vada (Quiviesa river)	ESPNNO14	43°5'22"N 4°40'30"W	L.F. Valladares	**LT602126**	**LT601856**					-	
50	*D. aubei sanfilippoi*	IBE-DV108	Spain	Cantabria, Bárago	ESPNNO15	ca. 43° 4'23"N 4°37'06"W	L.F. Valladares	**LT602127**	**LT601857**					-	
51	*D. aubei sanfilippoi*	IBE-DV109	Spain	Cantabria, Bárago	ESPNNO15	ca. 43° 4'23"N 4°37'06"W	L.F. Valladares	**LT602128**	**LT601858**					-	
52	*D. aubei sanfilippoi*	IBE-DV110	Spain	Cantabria, Vada (Vejo river)	ESPNNO16	43°5'24"N 4°40'33"W	L.F. Valladares	**LT602129**	**LT601859**	**LT602526**	**LT602351**	**LT602434**		NO	
53	*D. aubei sanfilippoi*	IBE-DV111	Spain	Cantabria, Vada (Vejo river)	ESPNNO16	43°5'24"N 4°40'33"W	L.F. Valladares	**LT602130**	**LT601860**					-	
54	*D. aubei sanfilippoi*	IBE-DV209	France	Languedoc-Rousillon, Pyrenees Orientales department, Prats de Mollo	FRAO1	ca. 42°26'36"N 2°27'41"E	H. Fery	**LT602131**	**LT601861**	**LT602527**	**LT602352**	**LT602435**	**LT602613**	YES	A
55	*D. aubei sanfilippoi*	IBE-DV210	France	Languedoc-Rousillon, Pyrenees Orientales department, Prats de Mollo	FRAO1	ca. 42°26'36"N 2°27'41"E	H. Fery	**LT602132**	**LT601862**					-	
56	*D. aubei sanfilippoi*	IBE-DV211	France	Languedoc-Rousillon, Pyrenees Orientales department, Prats de Mollo	FRAO1	ca. 42°26'36"N 2°27'41"E	H. Fery	**LT602133**	**LT601863**					-	
57	*D. aubei sanfilippoi*	IBE-DV212	France	Languedoc-Rousillon, Pyrenees Orientales department, Prats de Mollo	FRAO1	ca. 42°26'36"N 2°27'41"E	H. Fery	**LT602134**	**LT601864**					-	
58	*D. aubei sanfilippoi*	IBE-DV261	France	Languedoc-Rousillon, Pyrenees Orientales department, Prats de Mollo	FRAO1	ca. 42°26'36"N 2°27'41"E	H. Fery	**LT602135**	**LT601865**					-	
59	*D. aubei sanfilippoi*	NHM-ER38	France	Languedoc-Rousillon, Pyrenees Orientales department, Prats de Mollo	FRAO1	ca. 42°26'36"N 2°27'41"E	H. Fery		**LT601866**	**LT602528**				-	
60	*D. aubei sanfilippoi*	NHM-ER41	Spain	Castilla y León, León province, Puerto de San Glorio	ESPNNO3	ca. 43°3'57"N 4°45'31"W	D.T. Bilton		**LT601867**	**LT602529**				-	
61	*D. aubei spp.*	IBE-DV57	France	Languedoc-Rousillon, Gard province, Le Vigan	FRAO2	44º02'14.7"N 3º34'56.8"E	I. Ribera & A. Cieslak	**LT602136**	**LT601868**					-	
62	*D. aubei spp.*	IBE-DV58	France	Languedoc-Rousillon, Gard province, Le Vigan	FRAO2	44º02'14.7"N 3º34'56.8"E	I. Ribera & A. Cieslak	**LT602137**	**LT601869**					-	
63	*D. aubei spp.*	IBE-DV140	France	Auvergne, Cantal department, Vedrines-St. Loup	FRAO3	45°4'0.5"N 3°18'24.6"E	I. Ribera & A. Cieslak	**LT602138**	**LT601870**	**LT602530**	**LT602353**			-	
64	*D. aubei spp.*	IBE-DV141	France	Auvergne, Cantal department, Vedrines-St. Loup	FRAO3	45°4'0.5"N 3°18'24.6"E	I. Ribera & A. Cieslak	**LT602139**	**LT601871**	**LT602531**	**LT602354**	**LT602436**	**LT602614**	YES	B

65	*D. aubei spp.*	IBE-DV148	France	Languedoc-Rousillon, Gard province, Le Vigan	FRAO2	44º02'14.7"N 3º34'56.8"E	I. Ribera & A. Cieslak	**LT602140**	**LT601872**					-	
66	*D. aubei spp.*	IBE-DV149	France	Languedoc-Rousillon, Gard province, Le Vigan	FRAO2	44º02'14.7"N 3º34'56.8"E	I. Ribera & A. Cieslak	**LT602141**	**LT601873**	**LT602532**	**LT602355**	**LT602437**		-	
67	*D. aubei spp.*	IBE-DV160	France	Auvergne, Cantal department, Laveissière	FRAO4	45°6'55.3"N 2°48'47.4"E	I. Ribera & A. Cieslak	**LT602142**	**LT601874**	**LT602533**	**LT602356**	**LT602438**	**LT602615**	YES	B
68	*D. aubei spp.*	IBE-DV161	France	Auvergne, Cantal department, Laveissière	FRAO4	45°6'55.3"N 2°48'47.4"E	I. Ribera & A. Cieslak	**LT602143**	**LT601875**					-	
69	*D. aubei spp.*	IBE-DV162	France	Auvergne, Cantal department, Laveissière	FRAO4	45°6'55.3"N 2°48'47.4"E	I. Ribera & A. Cieslak	**LT602144**	**LT601876**					-	
70	*D. aubei spp.*	IBE-DV163	France	Auvergne, Cantal department, Laveissière	FRAO4	45°6'55.3"N 2°48'47.4"E	I. Ribera & A. Cieslak	**LT602145**	**LT601877**					-	
71	*D. aubei spp.*	IBE-DV164	France	Auvergne, Cantal department, Laveissière	FRAO4	45°6'55.3"N 2°48'47.4"E	I. Ribera & A. Cieslak	**LT602146**	**LT601878**					-	
72	*D. aubei spp.*	IBE-DV172	France	Auvergne, Cantal department, Vedrines-St. Loup	FRAO3	45°4'0.5"N 3°18'24.6"E	I. Ribera & A. Cieslak	**LT602147**	**LT601879**					-	
73	*D. aubei spp.*	IBE-DV173	France	Auvergne, Cantal department, Vedrines-St. Loup	FRAO3	45°4'0.5"N 3°18'24.6"E	I. Ribera & A. Cieslak	**LT602148**	**LT601880**					-	
74	*D. aubei spp.*	IBE-RA122	France	Languedoc-Rousillon, Gard province, Le Vigan	FRAO2	44º02'14.7"N 3º34'56.8"E	I. Ribera & A. Cieslak	**LT602149**	**LT601881**	**LT602534**	**LT602357**	**LT602439**		YES	undetermined
75	*D. brannanii*	IBE-DV38	Spain (Mallorca)	Ternelles	IMALL1	39º53'37.2"N 3º00'14.9"E	I. Ribera & A. Cieslak	**LT602150**	**LT601882**					-	
76	*D. brannanii*	IBE-DV39	Spain (Mallorca)	Ternelles	IMALL1	39º53'37.2"N 3º00'14.9"E	I. Ribera & A. Cieslak	**LT602151**	**LT601883**					-	
77	*D. brannanii*	IBE-DV40	Spain (Mallorca)	Sóller	IMALL2	39º45'45.7"N 2º42'39.3"E	I. Ribera & A. Cieslak	**LT602152**	**LT601884**	**LT602535**	**LT602358**	**LT602440**		NO	
78	*D. brannanii*	IBE-DV74	Spain (Mallorca)	Sóller	IMALL3	ca. 39°46'11"N 2°43'60"E	G. Wewalka	**LT602153**	**LT601885**	**LT602536**	**LT602359**	**LT602441**		-	
79	*D. brannanii*	IBE-DV75	Spain (Mallorca)	Sóller	IMALL3	ca. 39°46'11"N 2°43'60"E	G. Wewalka	**LT602154**	**LT601886**					-	
80	*D. brannanii*	IBE-DV76	Spain (Mallorca)	Sóller	IMALL3	ca. 39°46'11"N 2°43'60"E	G. Wewalka	**LT602155**	**LT601887**					-	
81	*D. brannanii*	IBE-DV120	Spain (Mallorca)	Ternelles	IMALL1	39º53'37.2"N 3º00'14.9"E	I. Ribera & A. Cieslak	**LT602156**	**LT601888**					-	
82	*D. brannanii*	IBE-DV121	Spain (Mallorca)	Ternelles	IMALL1	39º53'37.2"N 3º00'14.9"E	I. Ribera & A. Cieslak	**LT602157**	**LT601889**					-	
83	*D. brannanii*	IBE-RA313	Spain (Mallorca)	Sóller	IMALL3	ca. 39°46'11"N 2°43'60"E	G. Wewalka	**LT602158**	**LT601890**					-	
84	*D. brannanii*	IBE-RA314	Spain (Mallorca)	Sóller	IMALL3	ca. 39°46'11"N 2°43'60"E	G. Wewalka	**LT602159**	**LT601891**					-	
85	*D. brannanii*	MNCN-AI178	Spain (Mallorca)	Ternelles	IMALL1	39º53'37.2"N 3º00'14.9"E	I. Ribera & A. Cieslak	LN995094	HE610180	HF931404	LN995133	**LT602442**		NO	
86	*D. brannanii*	MNCN-AI296	Spain (Mallorca)	Sóller	IMALL2	39º45'45.7"N 2º42'39.3"E	I. Ribera & A. Cieslak	**LT602160**	**LT601892**					-	
87	*D. costipennis costipennis*	IBE-DV155	Portugal	Guarda district, Gouveia	PORCN8	40°29'49"N 7°35'56"W	J. Fresneda	**LT602161**	**LT601893**	**LT602537**	**LT602360**	**LT602443**		-	
88	*D. costipennis costipennis*	IBE-DV156	Portugal	Guarda district, Gouveia	PORCN8	40°29'49"N 7°35'56"W	J. Fresneda	**LT602162**	**LT601894**					-	
89	*D. costipennis costipennis*	IBE-DV157	Portugal	Guarda district, Gouveia	PORCN8	40°29'49"N 7°35'56"W	J. Fresneda	**LT602163**	**LT601895**					-	
90	*D. costipennis costipennis*	IBE-DV158	Portugal	Guarda district, Gouveia	PORCN8	40°29'49"N 7°35'56"W	J. Fresneda	**LT602164**	**LT601896**					-	
91	*D. costipennis costipennis*	IBE-DV204	Portugal	Guarda district, Gouveia	PORCN8	40°29'49"N 7°35'56"W	J. Fresneda	**LT602165**	**LT601897**					-	
92	*D. costipennis costipennis*	MNCN-AI183	Portugal	Guarda district, Manteigas (Serra da Estrela)	PORCN1	ca. 40º19'57"N 7º37'03"W	I. Ribera	LN995095	HE610181	LN995169	LN995134	**LT602444**		-	
93	*D. costipennis gignouxi*	IBE-DV19	Spain	Castilla y León, León province, Valverdín	ESPNNO13	ca. 42°56'53"N 5°32'10"W	L.F. Valladares	LN995096	LN995068	LN995170	LN995135	**LT602445**		NO	
94	*D. costipennis gignouxi*	NHM-ER40	Spain	Castilla y León, León province, Puerto de San Glorio	ESPNNO3	ca. 43°3'57"N 4°45'31"W	D.T. Bilton		AF309324	AY250951				-	
95	*D. delarouzei*	IBE-AF85	Spain	Aragón, Huesca province, Valle de Plan	ESPPIR1	ca. 42°34'39"N 0°20'7"E	I. Esteban		**LT601898**					-	
96	*D. delarouzei*	IBE-DV1	Spain	Cataluña, Barcelona province, Bagà	ESPPIR10	42º16'05.3"N 1º48"46.1"E	I. Ribera, P. Aguilera & C. Hernando	**LT602166**	**LT601899**	**LT602538**	**LT602361**			-	
97	*D. delarouzei*	IBE-DV2	Spain	Cataluña, Barcelona province, Bagà	ESPPIR10	42º16'05.3"N 1º48"46.1"E	I. Ribera, P. Aguilera & C. Hernando	**LT602167**	**LT601900**					-	
98	*D. delarouzei*	IBE-DV3	Spain	Cataluña, Lleida province, Llesp	ESPPIR12	42º27'24.5"N 0º44'57"E	I. Ribera, A. Cieslak & J. Fresneda	**LT602168**	**LT601901**	**LT602539**				-	
99	*D. delarouzei*	IBE-DV4	Spain	Cataluña, Lleida province, Llesp	ESPPIR12	42º27'24.5"N 0º44'57"E	I. Ribera, A. Cieslak & J. Fresneda	**LT602169**	**LT601902**					-	
100	*D. delarouzei*	IBE-DV5	Spain	Aragón, Huesca province, Bonansa	ESPPIR13	42º25'26.3"N 0º41'17.6"E	I. Ribera & A. Cieslak	**LT602170**	**LT601903**	**LT602540**	**LT602362**			-	
101	*D. delarouzei*	IBE-DV98	Spain	Aragón, Huesca province, Aragues del Puerto	ESPPIR7	42º45'16.93''N 0º37'59.65''W	I. Esteban	**LT602171**	**LT601904**	**LT602541**	**LT602363**	**LT602446**	**LT602616**	YES	A
102	*D. delarouzei*	IBE-DV105	Spain	Cataluña, Lleida province, Llesp	ESPPIR12	42º27'24.5"N 0º44'57"E	I. Ribera, A. Cieslak & J. Fresneda	**LT602172**	**LT601905**					-	
103	*D. delarouzei*	IBE-DV106	Spain	Cataluña, Lleida province, Llesp	ESPPIR12	42º27'24.5"N 0º44'57"E	I. Ribera, A. Cieslak & J. Fresneda	**LT602173**	**LT601906**					-	
104	*D. delarouzei*	IBE-DV107	Spain	Cataluña, Lleida province, Llesp	ESPPIR12	42º27'24.5"N 0º44'57"E	I. Ribera, A. Cieslak & J. Fresneda	**LT602174**	**LT601907**	**LT602542**	**LT602364**	**LT602447**	**LT602617**	YES	A
105	*D. delarouzei*	IBE-DV135	Spain	Cataluña, Girona province, Collada de Tosas	ESPPIR15	42º20'07.8"N 2º04'22.8E	I. Ribera & A. Cieslak	**LT602175**	**LT601908**	**LT602543**	**LT602365**	**LT602448**		-	
106	*D. delarouzei*	IBE-DV159	Spain	Cataluña, Lleida province, Rubió	ESPPIR20	42°22'42"N 1°13'27"E	I. Ribera & A. Cieslak	**LT602176**	**LT601909**	**LT602544**	**LT602366**	**LT602449**	**LT602618**	YES	A
107	*D. delarouzei*	IBE-DV175	Spain	Aragón, Huesca province, Santa Cruz de Serós	ESPPIR21	42°31'5.2"N 0°41'36.8"W	I. Ribera & A. Cieslak		**LT601910**	**LT602545**	**LT602367**	**LT602450**		-	
108	*D. delarouzei*	IBE-DV176	Spain	Aragón, Huesca province, Santa Cruz de Serós	ESPPIR21	42°31'5.2"N 0°41'36.8"W	I. Ribera & A. Cieslak	**LT602177**	**LT601911**					-	
109	*D. delarouzei*	IBE-DV177	Spain	Aragón, Huesca province, Santa Cruz de Serós	ESPPIR21	42°31'5.2"N 0°41'36.8"W	I. Ribera & A. Cieslak	**LT602178**	**LT601912**					-	
110	*D. delarouzei*	IBE-DV191	Spain	Aragón, Huesca province, Santa Cruz de Serós	ESPPIR21	42°31'5.2"N 0°41'36.8"W	I. Ribera & A. Cieslak	**LT602179**	**LT601913**					-	
111	*D. delarouzei*	IBE-DV192	Spain	Aragón, Huesca province, Santa Cruz de Serós	ESPPIR21	42°31'5.2"N 0°41'36.8"W	I. Ribera & A. Cieslak	**LT602180**	**LT601914**					-	
112	*D. delarouzei*	IBE-DV201	Spain	Aragón, Huesca province, Astún	ESPPIR18	ca. 42°48'27"N 0°30'47"W	I. Esteban	**LT602181**	**LT601915**	**LT602546**	**LT602368**	**LT602451**		-	
113	*D. delarouzei*	IBE-DV202	Spain	Aragón, Huesca province, Astún	ESPPIR18	ca. 42°48'27"N 0°30'47"W	I. Esteban	**LT602182**	**LT601916**					-	
114	*D. delarouzei*	IBE-RA337	Spain	Aragón, Huesca province, Aragues del Puerto	ESPPIR7	42º45'16.93''N 0º37'59.65''W	I. Esteban	LN995097	LN995069	LN995171	LN995136	**LT602452**		-	
115	*D. delarouzei*	MNCN-AI1071	Spain	Cataluña, Barcelona province, Montseny	ESPPIR2	41º45'23.5"N 2º24'49.8"E	I. Ribera, P. Aguilera & C. Hernando	**LT602183**	**LT601917**	**LT602547**	**LT602369**	**LT602453**	**LT602619**	YES	A
116	*D. delarouzei*	NHM-ER37	Spain	Cataluña, Lleida province, Llesp	ESPPIR3	ca. 42º27'24"N 0º44'57"E	H. Fery		**LT601918**	**LT602548**				-	
117	*D. delarouzei*	NHM-IR78	Spain	Cataluña, Barcelona province, Saldes	ESPPIR4	ca. 42°13'37"N 1°44'32"E	P. Aguilera		**LT601919**	**LT602549**				-	
118	*D. latus*	IBE-DV17	Scotland	Isle of Skye, Broadford river	SCO1	ca. 57°13'57"N 5°56'22"W	G.N. Foster	**LT602184**	**LT601920**	**LT602550**				-	
119	*D. latus*	IBE-DV18	Scotland	Isle of Skye, Broadford river	SCO1	ca. 57°13'57"N 5°56'22"W	G.N. Foster	**LT602185**	**LT601921**					-	
120	*D. latus*	IBE-DV45	Bulgaria	Kyustendil province, Rila	BUL3	ca. 42°7'55"N 23°8'40"E	D.T. Bilton	**LT602186**	**LT601922**	**LT602551**	**LT602370**	**LT602454**		-	
121	*D. latus*	IBE-DV46	Bulgaria	Kyustendil province, Rila	BUL3	ca. 42°7'55"N 23°8'40"E	D.T. Bilton	**LT602187**	**LT601923**					-	
122	*D. latus*	IBE-DV77	Croatia	Istria county, Momjan	CRO2	45º26'30.8"N 13º42'37.8"E	I. Ribera & A. Cieslak	**LT602188**	**LT601924**	**LT602552**	**LT602371**	**LT602455**		-	
123	*D. latus*	IBE-DV78	Croatia	Istria county, Momjan	CRO2	45º26'30.8"N 13º42'37.8"E	I. Ribera & A. Cieslak	**LT602189**	**LT601925**					-	
124	*D. latus*	IBE-DV80	Slovenia	Carniola, Cerkniščica	ESL2	45º45'57.5"N 14º21'40.0"E	I. Ribera & A. Cieslak	**LT602190**	**LT601926**	**LT602553**	**LT602372**	**LT602456**		NO	
125	*D. latus*	IBE-DV81	Slovenia	Carniola, Cerkniščica	ESL2	45º45'57.5"N 14º21'40.0"E	I. Ribera & A. Cieslak	**LT602191**	**LT601927**					-	
126	*D. latus*	IBE-DV82	Slovenia	Carniola, Cerkniščica	ESL2	45º45'57.5"N 14º21'40.0"E	I. Ribera & A. Cieslak	**LT602192**	**LT601928**					-	
127	*D. latus*	IBE-DV112	Scotland	Isle of Skye, Broadford river	SCO1	ca. 57°13'57"N 5°56'22"W	G.N. Foster	**LT602193**	**LT601929**	**LT602554**	**LT602373**	**LT602457**		NO	
128	*D. latus*	IBE-DV113	Scotland	Isle of Skye, Broadford river	SCO1	ca. 57°13'57"N 5°56'22"W	G.N. Foster	**LT602194**	**LT601930**					-	
129	*D. latus*	IBE-DV168	Croatia	Istria county, Momjan	CRO2	45º26'30.8"N 13º42'37.8"E	I. Ribera & A. Cieslak	**LT602195**	**LT601931**					-	
130	*D. latus*	IBE-DV169	Croatia	Istria county, Momjan	CRO2	45º26'30.8"N 13º42'37.8"E	I. Ribera & A. Cieslak		**LT601932**					-	
131	*D. latus*	IBE-DV182	Germany	Rheinland-Pfalz, river Flaunbach	ALE6	50°8'59.5"N 7°16'35.0"E	I. Ribera & A. Cieslak	**LT602196**	**LT601933**	**LT602555**	**LT602374**	**LT602458**		-	
132	*D. latus*	IBE-DV183	Germany	Rheinland-Pfalz, river Flaunbach	ALE6	50°8'59.5"N 7°16'35.0"E	I. Ribera & A. Cieslak	**LT602197**	**LT601934**					-	
133	*D. latus*	IBE-DV184	Germany	Rheinland-Pfalz, river Flaunbach	ALE6	50°8'59.5"N 7°16'35.0"E	I. Ribera & A. Cieslak		**LT601935**					-	
134	*D. latus*	IBE-DV189	Germany	Niedersachsen, Röhrsen	ALE5	52°48'26.1"N 10°37'57.9"E	I. Ribera & A. Cieslak	**LT602198**	**LT601936**	**LT602556**	**LT602375**	**LT602459**		-	
135	*D. latus*	IBE-DV190	Germany	Niedersachsen, Röhrsen	ALE5	52°48'26.1"N 10°37'57.9"E	I. Ribera & A. Cieslak	**LT602199**	**LT601937**					-	

136	*D. latus*	IBE-DV197	Germany	Rheinland-Pfalz, river Flaunbach	ALE6	50°8'59.5"N 7°16'35.0"E	I. Ribera & A. Cieslak	**LT602200**	**LT601938**					-	
137	*D. latus*	IBE-DV198	Germany	Rheinland-Pfalz, river Flaunbach	ALE6	50°8'59.5"N 7°16'35.0"E	I. Ribera & A. Cieslak	**LT602201**	**LT601939**					-	
138	*D. latus*	IBE-DV200	Germany	Niedersachsen, Röhrsen	ALE5	52°48'26.1"N 10°37'57.9"E	I. Ribera & A. Cieslak	**LT602202**	**LT601940**					-	
139	*D. latus*	IBE-RA343	Slovenia	Slovene Istria, Dernarnik	ESL1	45º29’13.8”N 13º50’01.7”E	I. Ribera, C. Hernando & A. Cieslak	LN995106	LN995074	LN995178	LN995144	**LT602460**		-	
140	*D. latus*	IBE-RA345	Croatia	Istria county, Momjan	CRO2	45º26’30.8”N 13º42’37.8”E	I. Ribera & A. Cieslak	**LT602203**	**LT601941**					-	
141	*D. latus*	IBE-RA350	Slovenia	Carniola, Cerkniščica	ESL2	45º45’57.5”N 14º21’40.0”E	I. Ribera & A. Cieslak	**LT602204**	**LT601942**					-	
142	*D. latus*	IBE-RA412	England	South East England, Hampshire county, New Forest	ENG1	ca. 50°50'24"N 1°37'20"W	I. Ribera	LN995107	LN995075	LN995179	LN995145	**LT602461**		-	
143	*D. latus*	IBE-RA413	England	South East England, Hampshire county, New Forest	ENG1	ca. 50°50'24"N 1°37'20"W	I. Ribera	**LT602205**	**LT601943**	**LT602557**				-	
144	*D. latus*	MNCN-AI137	Montenegro	Pljevlja region, river Vodenica	MON1	ca. 43°21'53"N 19°21'48"E	V. Pesic	**LT602206**	**LT601944**	**LT602558**	**LT602376**			-	
145	*D. latus*	MNCN-AI1038	Bulgaria	Kyustendil province, Rila	BUL3	ca. 42°7'55"N 23°8'40"E	D.T. Bilton	**LT602207**	**LT601945**					-	
146	*D. moestus inconspectus*	IBE-DV31	Italy (Sicily)	Mesina province, Cesaro-Randazzo	ISIC2	ca. 37°49'60"N 14°47'37"E	P. Abellán & F. Picazo	**LT602208**	**LT601946**					-	
147	*D. moestus inconspectus*	IBE-DV32	Italy (Sicily)	Mesina province, Cesaro-Randazzo	ISIC2	ca. 37°49'60"N 14°47'37"E	P. Abellán & F. Picazo	**LT602209**	**LT601947**					-	
148	*D. moestus inconspectus*	IBE-DV37	Italy (Sicily)	Catania province, Parco dei Nebrodi, river Flascio	ISIC5	ca. 37°54'13"N 14°52'52"E	P. Abellán & F. Picazo	**LT602210**	**LT601948**	**LT602559**	**LT602377**	**LT602462**		-	
149	*D. moestus inconspectus*	IBE-DV47	Italy	Veneto, Treviso province, Collalto	ITAN2	45º52’20.3”N 12º11’17.3”E	I. Ribera & A. Cieslak	**LT602211**	**LT601949**					-	
150	*D. moestus inconspectus*	IBE-DV48	Italy	Veneto, Treviso province, Collalto	ITAN2	45º52’20.3”N 12º11’17.3”E	I. Ribera & A. Cieslak	**LT602212**	**LT601950**					-	
151	*D. moestus inconspectus*	IBE-DV53	Greece	Epirus region, Kipoi	GRE1	39°51'42.2"N 20°47'10.4"E	P. & V. Ponel	**LT602213**	**LT601951**	**LT602560**	**LT602378**	**LT602463**	**LT602620**	YES	B
152	*D. moestus inconspectus*	IBE-DV54	Greece	Epirus region, Kipoi	GRE1	39°51'42.2"N 20°47'10.4"E	P. & V. Ponel	**LT602214**	**LT601952**					-	
153	*D. moestus inconspectus*	IBE-DV55	Greece	Pelopponese, Laconia region, Mistras	GRE2	ca. 37°4'25"N 22°22'27"E	G. Wewalka	**LT602215**	**LT601953**	**LT602561**	**LT602379**	**LT602464**		-	
154	*D. moestus inconspectus*	IBE-DV56	Greece	Pelopponese, Laconia region, Mistras	GRE2	ca. 37°4'25"N 22°22'27"E	G. Wewalka	**LT602216**	**LT601954**					-	
155	*D. moestus inconspectus*	IBE-DV62	Italy (Elba)	Porto Azurro, Madonna di Monserrato	IELBA1	ca. 42°47'6"N 10°23'30"E	M. Toledo	**LT602217**	**LT601955**	**LT602562**	**LT602380**	**LT602465**		NO	
156	*D. moestus inconspectus*	IBE-DV63	Italy (Elba)	Porto Azurro, Madonna di Monserrato	IELBA1	ca. 42°47'6"N 10°23'30"E	M. Toledo	**LT602218**	**LT601956**					-	
157	*D. moestus inconspectus*	IBE-DV64	Greece	Peloponnese, Achaia region, Erymanthos Mountains	GRE3	ca. 37°55'25"N 21°45'5"E	H. Fery & L. Hendrich	**LT602219**	**LT601957**	**LT602563**	**LT602381**	**LT602466**	**LT602621**	YES	B
158	*D. moestus inconspectus*	IBE-DV65	Greece	Peloponnese, Achaia region, Erymanthos Mountains	GRE3	ca. 37°55'25"N 21°45'5"E	H. Fery & L. Hendrich	**LT602220**	**LT601958**					-	
159	*D. moestus inconspectus*	IBE-DV79	Spain	Aragón, Zaragoza province, El Frago	ESPPIR6	42º 17'43,7''N 0º53'45,5''W	I. Esteban	LN995111	LN995079	LN995183	LN995149	**LT602467**		-	
160	*D. moestus inconspectus*	IBE-DV85	France	Provence-Alpes-Cote D´azur, Var department, Comps	FRAE4	43º42'56.6"N 6º33'03.7"E	I. Ribera & A. Cieslak	**LT602221**	**LT601959**					-	
161	*D. moestus inconspectus*	IBE-DV86	France	Provence-Alpes-Cote D´azur, Var department, Comps	FRAE4	43º42'56.6"N 6º33'03.7"E	I. Ribera & A. Cieslak	**LT602222**	**LT601960**				**LT602622**	YES	B
162	*D. moestus inconspectus*	IBE-DV87	France	Provence-Alpes-Cote D´azur, Var department, Comps	FRAE4	43º42'56.6"N 6º33'03.7"E	I. Ribera & A. Cieslak	**LT602223**	**LT601961**					-	
163	*D. moestus inconspectus*	IBE-DV94	Italy	Abruzzo, L´Aquila province, Mascioni	ITACN5	42º31’57”N 13º20’15”E	I. Ribera & A. Cieslak	**LT602224**	**LT601962**	**LT602564**	**LT602382**	**LT602468**		NO	
164	*D. moestus inconspectus*	IBE-DV95	Italy	Abruzzo, L´Aquila province, Mascioni	ITACN5	42º31’57”N 13º20’15”E	I. Ribera & A. Cieslak	**LT602225**	**LT601963**					-	
165	*D. moestus inconspectus*	IBE-DV96	Italy	Abruzzo, L´Aquila province, Mascioni	ITACN5	42º31’57”N 13º20’15”E	I. Ribera & A. Cieslak	**LT602226**	**LT601964**					-	
166	*D. moestus inconspectus*	IBE-DV118	Italy (Sicily)	Mesina province, Cesaro-Randazzo	ISIC2	ca. 37°49'60"N 14°47'37"E	P. Abellán & F. Picazo	**LT602227**	**LT601965**	**LT602565**	**LT602383**	**LT602469**	**LT602623**	YES	B
167	*D. moestus inconspectus*	IBE-DV119	Italy (Sicily)	Mesina province, Cesaro-Randazzo	ISIC2	ca. 37°49'60"N 14°47'37"E	P. Abellán & F. Picazo	**LT602228**	**LT601966**					-	
168	*D. moestus inconspectus*	IBE-DV126	Italy	Veneto, Treviso province, Collalto	ITAN2	45º52’20.3”N 12º11’17.3”E	I. Ribera & A. Cieslak	**LT602229**	**LT601967**					-	
169	*D. moestus inconspectus*	IBE-DV127	Italy	Veneto, Treviso province, Collalto	ITAN2	45º52’20.3”N 12º11’17.3”E	I. Ribera & A. Cieslak	**LT602230**	**LT601968**					-	
170	*D. moestus inconspectus*	IBE-DV128	Spain	Andalucia, Jaén province, Sierra de Cazorla	ESPSUR14	ca. 38°6'9"N 2°48'46"W	A. Castro	**LT602231**	**LT601969**	**LT602566**	**LT602384**	**LT602470**		NO	
171	*D. moestus inconspectus*	IBE-DV129	Spain	Andalucia, Jaén province, Sierra de Cazorla	ESPSUR14	ca. 38°6'9"N 2°48'46"W	A. Castro	**LT602232**	**LT601970**					-	
172	*D. moestus inconspectus*	IBE-DV132	Greece	Pelopponese, Laconia region, Mistras	GRE2	ca. 37°4'25"N 22°22'27"E	G. Wewalka	**LT602233**	**LT601971**					-	
173	*D. moestus inconspectus*	IBE-DV133	Greece	Pelopponese, Laconia region, Mistras	GRE2	ca. 37°4'25"N 22°22'27"E	G. Wewalka	**LT602234**	**LT601972**					-	
174	*D. moestus inconspectus*	IBE-DV142	Spain	Aragón, Huesca province, Acumuer	ESPPIR17	42º37'34,65''N 0º24'0,62''W	I. Esteban	**LT602235**	**LT601973**	**LT602567**	**LT602385**	**LT602471**		-	
175	*D. moestus inconspectus*	IBE-DV143	Spain	Aragón, Huesca province, Acumuer	ESPPIR17	42º37'34,65''N 0º24'0,62''W	I. Esteban	**LT602236**	**LT601974**					-	
176	*D. moestus inconspectus*	IBE-DV146	Spain	Aragón, Teruel province, Aliaga	ESPEST4	ca. 40°40'53"N 0°41'5"W	I. Esteban	**LT602237**	**LT601975**					-	
177	*D. moestus inconspectus*	IBE-DV147	Spain	Aragón, Teruel province, Aliaga	ESPEST4	ca. 40°40'53"N 0°41'5"W	I. Esteban	**LT602238**	**LT601976**					-	
178	*D. moestus inconspectus*	IBE-DV178	Spain	Aragón, Huesca province, Santa Cruz de Serós	ESPPIR21	42°31'5.2"N 0°41'36.8"W	I. Ribera & A. Cieslak	**LT602239**	**LT601977**	**LT602568**	**LT602386**	**LT602472**		-	
179	*D. moestus inconspectus*	IBE-DV179	Spain	Aragón, Huesca province, Santa Cruz de Serós	ESPPIR21	42°31'5.2"N 0°41'36.8"W	I. Ribera & A. Cieslak	**LT602240**	**LT601978**					-	
180	*D. moestus inconspectus*	IBE-DV180	Spain	Aragón, Huesca province, Bernués	ESPPIR22	42°26'38"N 0°36'51"W	I. Ribera & A. Cieslak	**LT602241**	**LT601979**	**LT602569**	**LT602387**	**LT602473**		NO	
181	*D. moestus inconspectus*	IBE-DV181	Spain	Aragón, Huesca province, Bernués	ESPPIR22	42°26'38"N 0°36'51"W	I. Ribera & A. Cieslak		**LT601980**					-	
182	*D. moestus inconspectus*	IBE-DV186	Spain	Aragón, Huesca province, Acumuer	ESPPIR17	42º37'34,65''N 0º24'0,62''W	I. Esteban	**LT602242**	**LT601981**					-	
183	*D. moestus inconspectus*	IBE-DV187	Spain	Aragón, Huesca province, Acumuer	ESPPIR17	42º37'34,65''N 0º24'0,62''W	I. Esteban	**LT602243**	**LT601982**					-	
184	*D. moestus inconspectus*	IBE-DV188	Spain	Aragón, Teruel province, Aliaga	ESPEST4	ca. 40°40'53"N 0°41'5"W	I. Esteban	**LT602244**	**LT601983**	**LT602570**	**LT602388**	**LT602474**	**LT602624**	YES	A
185	*D. moestus inconspectus*	IBE-DV193	Spain	Aragón, Huesca province, Santa Cruz de Serós	ESPPIR21	42°31'5.2"N 0°41'36.8"W	I. Ribera & A. Cieslak	**LT602245**	**LT601984**					-	
186	*D. moestus inconspectus*	IBE-DV194	Spain	Aragón, Huesca province, Bernués	ESPPIR22	42°26'38"N 0°36'51"W	I. Ribera & A. Cieslak	**LT602246**	**LT601985**					-	
187	*D. moestus inconspectus*	IBE-DV195	Spain	Aragón, Huesca province, Bernués	ESPPIR22	42°26'38"N 0°36'51"W	I. Ribera & A. Cieslak		**LT601986**					-	
188	*D. moestus inconspectus*	IBE-DV196	Spain	Aragón, Huesca province, Bernués	ESPPIR22	42°26'38"N 0°36'51"W	I. Ribera & A. Cieslak	**LT602247**	**LT601987**					-	
189	*D. moestus inconspectus*	IBE-DV199	Spain	Aragón, Huesca province, Acumuer	ESPPIR17	42º37'34,65''N 0º24'0,62''W	I. Esteban	**LT602248**	**LT601988**					-	
190	*D. moestus inconspectus*	IBE-DV203	Spain	Aragón, Huesca province, Ansó	ESPPIR19	ca. 42°45'39"N 0°49'49"W	I. Esteban	**LT602249**	**LT601989**	**LT602571**	**LT602389**	**LT602475**	**LT602625**	YES	A
191	*D. moestus inconspectus*	IBE-DV205	Italy (Sicily)	Palermo province, Parco naturalle delle Madonie	ISIC6	ca. 37°53'27"N 14°0'1"E	P. Abellán & F. Picazo	**LT602250**	**LT601990**	**LT602572**	**LT602390**	**LT602476**	**LT602626**	YES	B
192	*D. moestus inconspectus*	IBE-DV206	Italy (Sicily)	Palermo province, Parco naturalle delle Madonie	ISIC6	ca. 37°53'27"N 14°0'1"E	P. Abellán & F. Picazo	**LT602251**	**LT601991**					-	
193	*D. moestus inconspectus*	IBE-DV207	Italy (Sicily)	Palermo province, Parco naturalle delle Madonie	ISIC6	ca. 37°53'27"N 14°0'1"E	P. Abellán & F. Picazo	**LT602252**	**LT601992**					-	
194	*D. moestus inconspectus*	IBE-DV208	Italy (Sicily)	Palermo province, Parco naturalle delle Madonie	ISIC6	ca. 37°53'27"N 14°0'1"E	P. Abellán & F. Picazo	**LT602253**	**LT601993**					-	
195	*D. moestus inconspectus*	IBE-DV260	Italy (Sicily)	Palermo province, Parco naturalle delle Madonie	ISIC6	ca. 37°53'27"N 14°0'1"E	P. Abellán & F. Picazo	**LT602254**	**LT601994**					-	
196	*D. moestus inconspectus*	IBE-DV263	Italy (Sicily)	Mesina province, Cesaro-Randazzo	ISIC2	ca. 37°49'60"N 14°47'37"E	P. Abellán & F. Picazo	**LT602255**	**LT601995**					-	
197	*D. moestus inconspectus*	IBE-RA145	Italy (Elba)	Porto Azurro, Madonna di Monserrato	IELBA1	ca. 42°47'6"N 10°23'30"E	M. Toledo	**LT602256**	**LT601996**					-	
198	*D. moestus inconspectus*	IBE-RA213	Greece	Peloponnese, Achaia region, Erymanthos Mountains	GRE3	ca. 37°55'25"N 21°45'5"E	H. Fery & L. Hendrich	**LT602257**	**LT601997**					-	
199	*D. moestus inconspectus*	IBE-RA331	Spain	Cataluña, Lleida province, Llinars	ESPPIR5	ca. 42°6'12"N 1°41'24"E	J. Fresneda	**LT602258**	**LT601998**	**LT602573**	**LT602391**	**LT602477**	**LT602627**	YES	A
200	*D. moestus inconspectus*	IBE-RA333	Croatia	Istria county, Vranja	CRO1	ca. 45°19'7"N 14°8'21"E	H. Fery		**LT601999**	**LT602574**	**LT602392**	**LT602478**		NO	
201	*D. moestus inconspectus*	IBE-RA336	Spain	Aragón, Zaragoza province, El Frago	ESPPIR6	42º 17'43,7''N 0º53'45,5''W	I. Esteban	**LT602259**	**LT602000**					-	
202	*D. moestus inconspectus*	IBE-RA338	Spain	Aragón, Huesca province, Aragues del Puerto	ESPPIR7	42º45'16.93''N 0º37'59.65''W	I. Esteban	**LT602260**	**LT602001**	**LT602575**	**LT602393**	**LT602479**		-	
203	*D. moestus inconspectus*	IBE-RA365	Morocco	Meknès-Tafilalet, Khénifra Province, Kerrouchen (Middle Atlas)	MARATL9	32º48'22.1"N 5º16'13.9"W	I. Ribera, P. Aguilera & C. Hernando	**LT602261**	**LT602002**	**LT602576**	**LT602394**	**LT602480**		NO	
204	*D. moestus inconspectus*	IBE-RA374	France	Provence-Alpes-Cote D´azur, Var department, Comps	FRAE4	43º42'56.6"N 6º33'03.7"E	I. Ribera & A. Cieslak	**LT602262**	**LT602003**	**LT602577**	**LT602395**	**LT602481**	**LT602628**	YES	B
205	*D. moestus inconspectus*	IBE-RA429	Italy	Abruzzo, L´Aquila province, Mascioni	ITACN5	42º31’57”N 13º20’15”E	I. Ribera & A. Cieslak	**LT602263**	**LT602004**					-	
206	*D. moestus inconspectus*	IBE-RA430	Italy	Abruzzo, L´Aquila province, Mascioni	ITACN5	42º31’57”N 13º20’15”E	I. Ribera & A. Cieslak	**LT602264**	**LT602005**					NO	

207	*D. moestus inconspectus*	MNCN-AC37	Italy (Sicily)	Mesina province, Cesaro-Randazzo	ISIC2	ca. 37°49'60"N 14°47'37"E	P. Abellán & F. Picazo		**LT602006**	**LT602578**				-	
208	*D. moestus inconspectus*	MNCN-AH182	Italy (Sicily)	Catania province, Parco dei Nebrodi, river Flascio	ISIC5	ca. 37°54'13"N 14°52'52"E	P. Abellán & F. Picazo	**LT602265**	**LT602007**					-	
209	*D. moestus inconspectus*	MNCN-AI672	Bulgaria	Blagoevgrad province, Filipovo (Rhodope Mountains)	BUL1	ca. 41°45'53"N 23°41'22"E	D.T. Bilton	LN995108	LN995076	LN995180	LN995146	**LT602482**		-	
210	*D. moestus inconspectus*	MNCN-AI894	Spain	Andalucia, Jaén province, Hornos del Segura	ESPSUR2	ca. 38°14'6"N 2°43'42"W	A.G. Valdecasas	**LT602266**	**LT602008**	**LT602579**	**LT602396**	**LT602483**		NO	
211	*D. moestus inconspectus*	MNCN-AI937	Morocco	Sus-Masa-Draa, Ouarzazate province, Tachokchte	MARATL1	ca. 30°47'36"N 7°31'27"W	I. Ribera & A. Cieslak	LN995109	LN995077	LN995181	LN995147	**LT602484**		NO	
212	*D. moestus inconspectus*	MNCN-AI1048	Italy	Veneto, Treviso province, Collalto	ITAN2	45º52'20.3"N 12º11'17.3"E	I. Ribera & A. Cieslak	**LT602267**	**LT602009**	**LT602580**	**LT602397**	**LT602485**	**LT602629**	YES	A
213	*D. moestus inconspectus*	MNCN-AI1090	Spain	Andalucia, Jaén province, Sierra de Cazorla	ESPSUR14	ca. 38°6'9"N 2°48'46"W	A. Castro	**LT602268**	**LT602010**					-	
214	*D. moestus inconspectus*	MNCN-AI1202	Greece	Epirus region, Kipoi	GRE1	39°51'42.2"N 20°47'10.4"E	P. & V. Ponel	**LT602269**	**LT602011**					-	
215	*D. moestus inconspectus*	MNCN-AI1218	Greece	Pelopponese, Laconia region, Mistras	GRE2	ca. 37°4'25"N 22°22'27"E	G. Wewalka	**LT602270**	**LT602012**					-	
216	*D. moestus inconspectus*	NHM-IR82	Spain	Cataluña, Barcelona province, Saldes	ESPPIR4	ca. 42°13'37"N 1°44'32"E	P. Aguilera		**LT602013**	**LT602581**				-	
217	*D. moestus inconspectus*	NHM-IR207	Spain	Castilla La Mancha, Albacete province, river Endrinales	ESPSUR5	ca. 38°33'31"N 2°20'35"W	I. Ribera		**LT602014**					-	
218	*D. moestus inconspectus*	NHM-IR235	Morocco	Sus-Masa-Draa, Chtouka Ait Baha province, Ait-Iftene	MARATL4	ca. 30°4'10"N 9°9'26"W	I. Ribera		**LT602015**	**LT602582**				-	
219	*D. moestus inconspectus*	NHM-IR303	Italy	Lombardia, Pavia province, San Ponzo	ITAN4	ca. 44°50'23"N 9° 6'23"E	I. Ribera & A. Cieslak		**LT602016**	**LT602583**				-	
220	*D. moestus moestus*	IBE-DV41	Italy (Sardinia)	Ogliastra province, Codula di Luna	ICER1	40º10'16.0"N 9º33'36.7"E	P. & V. Ponel	**LT602271**	**LT602017**	**LT602584**	**LT602398**	**LT602486**		YES	undetermined
221	*D. moestus moestus*	IBE-DV42	Italy (Sardinia)	Ogliastra province, Codula di Luna	ICER1	40º10'16.0"N 9º33'36.7"E	P. & V. Ponel	**LT602272**	**LT602018**					-	
222	*D. moestus moestus*	IBE-DV66	Italy (Sardinia)	Ogliastra province, Villagrande Strisaili	ICER2	39°57'3.17"N 9°31'8.25"E	H. Fery & M. Toledo	**LT602273**	**LT602019**	**LT602585**	**LT602399**	**LT602487**		-	
223	*D. moestus moestus*	IBE-DV69	Italy (Sardinia)	Olbia-Tempio province, Monte Limbara	ICER3	40°51'30.18"N 9° 7'20.81"E	H. Fery & M. Toledo	LN995110	LN995078	LN995182	LN995148	**LT602488**		-	
224	*D. moestus moestus*	IBE-DV70	Italy (Sardinia)	Olbia-Tempio province, Monte Limbara	ICER3	40°51'30.18"N 9° 7'20.81"E	H. Fery & M. Toledo	**LT602274**	**LT602020**					-	
225	*D. moestus moestus*	IBE-DV71	Italy (Sardinia)	Olbia-Tempio province, Monte Limbara	ICER3	40°51'30.18"N 9° 7'20.81"E	H. Fery & M. Toledo	**LT602275**	**LT602021**					-	
226	*D. moestus moestus*	IBE-DV72	Italy (Sardinia)	Ogliastra province, Tortoli	ICER4	ca. 39°56'23"N 9°35'34"E	H. Fery & M. Toledo	**LT602276**	**LT602022**	**LT602586**	**LT602400**	**LT602489**		-	undetermined
227	*D. moestus moestus*	IBE-DV73	Italy (Sardinia)	Ogliastra province, Tortoli	ICER4	ca. 39°56'23"N 9°35'34"E	H. Fery & M. Toledo	**LT602277**	**LT602023**					-	
228	*D. moestus moestus*	IBE-DV122	Italy (Sardinia)	Ogliastra province, Codula di Luna	ICER1	40º10'16.0"N 9º33'36.7"E	P. & V. Ponel	**LT602278**	**LT602024**					-	
229	*D. moestus moestus*	IBE-DV123	Italy (Sardinia)	Ogliastra province, Codula di Luna	ICER1	40º10'16.0"N 9º33'36.7"E	P. & V. Ponel	**LT602279**	**LT602025**					-	
230	*D. moestus moestus*	IBE-DV166	Italy (Sardinia)	Ogliastra province, Tortoli	ICER4	ca. 39°56'23"N 9°35'34"E	H. Fery & M. Toledo	**LT602280**	**LT602026**					-	
231	*D. moestus moestus*	IBE-DV167	Italy (Sardinia)	Ogliastra province, Tortoli	ICER4	ca. 39°56'23"N 9°35'34"E	H. Fery & M. Toledo	**LT602281**	**LT602027**					-	
232	*D. moestus moestus*	IBE-RA307	Italy (Sardinia)	Ogliastra province, Villagrande Strisaili	ICER2	39°57'3.17"N 9°31'8.25"E	H. Fery & M. Toledo		**LT602028**					-	
233	*D. moestus moestus*	IBE-RA310	Italy (Sardinia)	Olbia-Tempio province, Monte Limbara	ICER3	40°51'30.18"N 9° 7'20.81"E	H. Fery & M. Toledo	**LT602282**	**LT602029**					-	
234	*D. moestus moestus*	IBE-RA311	Italy (Sardinia)	Ogliastra province, Tortoli	ICER4	ca. 39°56'23"N 9°35'34"E	H. Fery & M. Toledo	**LT602283**	**LT602030**					-	
235	*D. moestus moestus*	MNCN-AI886	Italy (Sardinia)	Ogliastra province, Codula di Luna	ICER1	40º10'16.0"N 9º33'36.7"E	P. & V. Ponel	**LT602284**	**LT602031**					-	
236	*D. moestus moestus*	NHM-IR156	France (Corsica)	Haute-Corse department, Vizzavona	ICOR1	ca. 42°6'59"N 9°6'40"E	I. Ribera & A. Cieslak	LN995105	AF309316	AF309259				-	
237	*D. moestus moestus*	NHM-MsC00B	France (Corsica)	Corse-du-Sud department, L´Ospedale	ICOR3	ca. 41°39'14"N 9°12'41"E	I. Ribera & A. Cieslak		**LT602032**	**LT602587**				-	
238	*D. platynotus*	IBE-DV137	Germany	Bayern, Schwaben region, Wertach	ALE2	47°35'9.6"N 10°22'52.2"E	L. Hendrich & R. Müller	**LT602285**	**LT602033**	**LT602588**	**LT602401**	**LT602490**		NO	
239	*D. platynotus*	IBE-DV213	Romania	Bacău county, Scutaru	RUM1	ca. 46°3'54"N 26°31'48"E	H. Fery	**LT602286**	**LT602034**	**LT602589**	**LT602402**	**LT602491**		-	
240	*D. platynotus*	IBE-RA316	Montenegro	Savnik	MON2	ca. 42°57'28"N 19°5'54"E	D.T. Bilton	**LT602287**	**LT602035**	**LT602590**	**LT602403**	**LT602492**		-	
241	*D. platynotus*	IBE-RA332	Germany	Saxonia, Dresden region, Pöbelbach	ALE1	50°48'57.96"N 13°39'44.14"E	L. Hendrich & R. Müller	**LT602288**	**LT602036**	**LT602591**				-	
242	*D. platynotus*	IBE-RA335	Macedonia	Ohrid district, Recica	MAC2	ca. 41°11'52"N 20°55'47"E	D.T. Bilton	**LT602289**	**LT602037**	**LT602592**	**LT602404**	**LT602493**		NO	
243	*D. platynotus*	IBE-RA445	Bulgaria	Kyustendil province, Rila	BUL3	ca. 42°7'55"N 23°8'40"E	D.T. Bilton	**LT602290**	**LT602038**					-	
244	*D. platynotus*	IBE-RA446	Bulgaria	Kyustendil province, Rila	BUL3	ca. 42°7'55"N 23°8'40"E	D.T. Bilton	**LT602291**	**LT602039**					-	
245	*D. platynotus*	IBE-RA447	Czech Republic	Central Bohemia region, Rakovnik district, Skryje	RCHE1	ca. 49°58'21"N 13°45'55"E	J. Statszny	**LT602292**	**LT602040**					-	
246	*D. platynotus*	IBE-RA448	Czech Republic	Central Bohemia region, Rakovnik district, Skryje	RCHE1	ca. 49°58'21"N 13°45'55"E	J. Statszny	**LT602293**	**LT602041**					-	
247	*D. platynotus*	MNCN-AI1039	Bulgaria	Kyustendil province, Rila	BUL3	ca. 42°7'55"N 23°8'40"E	D.T. Bilton	LN995115	HE610190	LN995186	LN995154	**LT602494**		-	
248	*D. platynotus*	MNCN-AI1121	Macedonia	Bitola district, Pelister National Park, Golemo Ezero	MAC1	ca. 40°58'9"N 21°12'19"E	R. B. Angus	**LT602294**	**LT602042**	**LT602593**	**LT602405**	**LT602495**		-	
249	*D. platynotus*	MNCN-AI1122	Germany	Saxonia, Dresden region, Pöbelbach	ALE1	50°48'57.96"N 13°39'44.14"E	L. Hendrich & R. Müller	LN995116	HE610191	HF931361	LN995155	**LT602496**		-	
250	*D. platynotus*	MNCN-AI1123	Germany	Saxonia, Dresden region, Pöbelbach	ALE1	50°48'57.96"N 13°39'44.14"E	L. Hendrich & R. Müller	**LT602295**	**LT602043**					-	
251	*D. platynotus*	MNCN-AI1275	Czech Republic	Central Bohemia region, Rakovnik district, Skryje	RCHE1	ca. 49°58'21"N 13°45'55"E	J. Statszny	**LT602296**	**LT602044**	**LT602594**	**LT602406**			-	
252	*D. platynotus*	NHM-IR246	Czech Republic	Central Bohemia region, Rakovnik district, Skryje	RCHE1	ca. 49°58'21"N 13°45'55"E	J. Statszny			**LT602595**				-	
253	*D. semirufus*	IBE-AF94	Italy	Emilia-Romagna, Modena province, Fanano	ITAN1	ca. 44°9'58"N 10°43'55"E	M. Toledo		**LT602045**	**LT602596**	**LT602407**	**LT602497**		-	
254	*D. semirufus*	IBE-DV29	Italy (Sicily)	Mesina province, Parco dei Nebrodi, Caserma Mafauda	ISIC1	ca. 37°53'42"N 14°30'31"E	P. Abellán & F. Picazo	**LT602297**	**LT602046**					-	
255	*D. semirufus*	IBE-DV30	Italy (Sicily)	Mesina province, Parco dei Nebrodi, Caserma Mafauda	ISIC1	ca. 37°53'42"N 14°30'31"E	P. Abellán & F. Picazo	**LT602298**	**LT602047**					-	
256	*D. semirufus*	IBE-DV33	Italy	Emilia-Romagna, Modena province, Fanano	ITAN1	ca. 44°9'58"N 10°43'55"E	M. Toledo	**LT602299**	**LT602048**					-	
257	*D. semirufus*	IBE-DV34	Italy (Sicily)	Mesina province, Parco dei Nebrodi, river Torti	ISIC4	ca. 37°53'38"N 14°39'6"E	P. Abellán & F. Picazo	**LT602300**	**LT602049**	**LT602597**	**LT602408**	**LT602498**	**LT602630**	YES	A
258	*D. semirufus*	IBE-DV35	Italy (Sicily)	Mesina province, Parco dei Nebrodi, river Torti	ISIC4	ca. 37°53'38"N 14°39'6"E	P. Abellán & F. Picazo	**LT602301**	**LT602050**					-	
259	*D. semirufus*	IBE-DV36	Italy (Sicily)	Mesina province, Parco dei Nebrodi, river Torti	ISIC4	ca. 37°53'38"N 14°39'6"E	P. Abellán & F. Picazo	**LT602302**	**LT602051**					-	
260	*D. semirufus*	IBE-DV51	France	Provence-Alpes-Cote D´azur, Alpes-Maritimes department, Monti	FRAE1	ca. 43°49'28"N 7°28'56"E	D.T. Bilton	**LT602303**	**LT602052**	**LT602598**	**LT602409**	**LT602499**		YES	undetermined
261	*D. semirufus*	IBE-DV52	France	Provence-Alpes-Cote D´azur, Alpes-Maritimes department, Monti	FRAE1	ca. 43°49'28"N 7°28'56"E	D.T. Bilton	**LT602304**	**LT602053**					-	
262	*D. semirufus*	IBE-DV59	Italy	Marche, Ascoli-Piceno province, Acquasanta	ITACN1	ca. 42°43'29"N 13°22'58"E	M. Toledo	**LT602305**	**LT602054**	**LT602599**	**LT602410**	**LT602500**	**LT602631**	YES	A
263	*D. semirufus*	IBE-DV88	Italy	Toscana, Arezzo province, Badia Prataglia	ITACN3	43º47'51"N 11º53'37"E	I. Ribera & A. Cieslak	**LT602306**	**LT602055**	**LT602600**	**LT602411**	**LT602501**		-	
264	*D. semirufus*	IBE-DV89	Italy	Toscana, Arezzo province, Badia Prataglia	ITACN3	43º47'51"N 11º53'37"E	I. Ribera & A. Cieslak	**LT602307**	**LT602056**					-	
265	*D. semirufus*	IBE-DV90	Italy	Toscana, Arezzo province, Badia Prataglia	ITACN3	43º47'51"N 11º53'37"E	I. Ribera & A. Cieslak	**LT602308**	**LT602057**					-	
266	*D. semirufus*	IBE-DV91	Italy	Toscana, Arezzo province, Fosso de Camaldoli	ITACN4	43º49'05"N 11º48'23"E	I. Ribera & A. Cieslak	**LT602309**	**LT602058**	**LT602601**				-	
267	*D. semirufus*	IBE-DV92	Italy	Toscana, Arezzo province, Fosso de Camaldoli	ITACN4	43º49'05"N 11º48'23"E	I. Ribera & A. Cieslak	**LT602310**	**LT602059**					-	
268	*D. semirufus*	IBE-DV93	Italy	Toscana, Arezzo province, Fosso de Camaldoli	ITACN4	43º49'05"N 11º48'23"E	I. Ribera & A. Cieslak	**LT602311**	**LT602060**	**LT602602**	**LT602412**	**LT602502**		YES	undetermined
269	*D. semirufus*	IBE-DV99	Italy	Abruzzo, L´Aquila province, Pescasseroli (Abruzzo N.P)	ITACN6	41°51'4.72"N 13°46'37.24"E	I. Ribera & A. Cieslak	**LT602312**	**LT602061**	**LT602603**	**LT602413**	**LT602503**	**LT602632**	YES	B
270	*D. semirufus*	IBE-DV100	Italy	Abruzzo, L´Aquila province, Pescasseroli (Abruzzo N.P)	ITACN6	41°51'4.72"N 13°46'37.24"E	I. Ribera & A. Cieslak	**LT602313**	**LT602062**					-	
271	*D. semirufus*	IBE-DV117	Italy (Sicily)	Mesina province, Parco dei Nebrodi, Caserma Mafauda	ISIC1	ca. 37°53'42"N 14°30'31"E	P. Abellán & F. Picazo	**LT602314**	**LT602063**	**LT602604**	**LT602414**	**LT602504**	**LT602633**	YES	A
272	*D. semirufus*	IBE-DV130	France	Provence-Alpes-Cote D´azur, Alpes-Maritimes department, Monti	FRAE1	ca. 43°49'28"N 7°28'56"E	D.T. Bilton	**LT602315**	**LT602064**					-	
273	*D. semirufus*	IBE-DV131	France	Provence-Alpes-Cote D´azur, Alpes-Maritimes department, Monti	FRAE1	ca. 43°49'28"N 7°28'56"E	D.T. Bilton	**LT602316**	**LT602065**					-	
274	*D. semirufus*	IBE-DV262	Italy (Sicily)	Mesina province, Parco dei Nebrodi, Caserma Mafauda	ISIC1	ca. 37°53'42"N 14°30'31"E	P. Abellán & F. Picazo	**LT602317**	**LT602066**					-	
275	*D. semirufus*	IBE-RA131	Italy	Marche, Ascoli-Piceno province, Acquasanta	ITACN1	ca. 42°43'29"N 13°22'58"E	M. Toledo	**LT602318**	**LT602067**					-	
276	*D. semirufus*	IBE-RA136	Italy	Emilia-Romagna, Modena province, Passo della Croce Arcana	ITAN6	ca. 44°8'4"N 10°46'55"E	M. Toledo	LN995118	LN995082	LN995188	LN995157	**LT602505**		-	
277	*D. semirufus*	IBE-RA340	Italy (Sicily)	Mesina province, Parco dei Nebrodi, Caserma Mafauda	ISIC7	ca. 37°53'31"N 14°31'35"E	P. Abellán & F. Picazo	**LT602319**	**LT602068**					-	

278	*D. semirufus*	IBE-RA406	Italy	Toscana, Arezzo province, Badia Prataglia	ITACN3	43º47’51”N 11º53’37”E	I. Ribera & A. Cieslak	**LT602320**	**LT602069**					-	
279	*D. semirufus*	IBE-RA407	Italy	Toscana, Arezzo province, Badia Prataglia	ITACN3	43º47’51”N 11º53’37”E	I. Ribera & A. Cieslak	**LT602321**	**LT602070**					-	
280	*D. semirufus*	IBE-RA417	Italy	Toscana, Arezzo province, Fosso de Camaldoli	ITACN4	43º49’05”N 11º48’23”E	I. Ribera & A. Cieslak	**LT602322**	**LT602071**					-	
281	*D. semirufus*	IBE-RA418	Italy	Toscana, Arezzo province, Fosso de Camaldoli	ITACN4	43º49’05”N 11º48’23”E	I. Ribera & A. Cieslak	**LT602323**	**LT602072**					-	
282	*D. semirufus*	IBE-RA427	Italy	Abruzzo, L´Aquila province, Mascioni	ITACN5	42º31’57”N 13º20’15”E	I. Ribera & A. Cieslak	**LT602324**	**LT602073**	**LT602605**	**LT602415**	**LT602506**		-	
283	*D. semirufus*	IBE-RA428	Italy	Abruzzo, L´Aquila province, Mascioni	ITACN5	42º31’57”N 13º20’15”E	I. Ribera & A. Cieslak	**LT602325**	**LT602074**					-	
284	*D. semirufus*	IBE-RA450	Italy	Abruzzo, L´Aquila province, Pescasseroli (Abruzzo N.P)	ITACN6	41°51'4.72"N 13°46'37.24"E	I. Ribera & A. Cieslak	**LT602326**	**LT602075**					-	
285	*D. semirufus*	IBE-RA451	Italy	Abruzzo, L´Aquila province, Pescasseroli (Abruzzo N.P)	ITACN6	41°51'4.72"N 13°46'37.24"E	I. Ribera & A. Cieslak	**LT602327**	**LT602076**					-	
286	*D. semirufus*	IBE-RA452	Italy	Abruzzo, L´Aquila province, Pescasseroli (Abruzzo N.P)	ITACN6	41°51'4.72"N 13°46'37.24"E	I. Ribera & A. Cieslak	**LT602328**	**LT602077**					-	
287	*D. semirufus*	MNCN-AC33	Italy (Sicily)	Mesina province, Parco dei Nebrodi, Caserma Mafauda	ISIC1	ca. 37°53'42"N 14°30'31"E	P. Abellán & F. Picazo		**LT602078**	**LT602606**				-	
288	*D. semirufus*	MNCN-AH178	Italy (Sicily)	Mesina province, Parco dei Nebrodi, lake of Biviere	ISIC3	ca. 37°56'29"N 14°40'15"E	P. Abellán & F. Picazo	**LT602329**	**LT602079**	**LT602607**	**LT602416**	**LT602507**		-	
289	*D. semirufus*	MNCN-AH179	Italy (Sicily)	Mesina province, Parco dei Nebrodi, river Torti	ISIC4	ca. 37°53'38"N 14°39'6"E	P. Abellán & F. Picazo		**LT602080**					-	
290	*D. semirufus*	MNCN-AI1114	France	Provence-Alpes-Cote D´azur, Alpes-Maritimes department, Monti	FRAE1	ca. 43°49'28"N 7°28'56"E	D.T. Bilton	**LT602330**	**LT602081**					-	
291	*D. semirufus*	NHM-IR299	France	Provence-Alpes-Cote D´azur, Alpes-Maritimes department, Monti	FRAE2	ca. 43°49'28"N 7°28'56"E	I. Ribera & A. Cieslak		**LT602082**	**LT602608**				-	
292	*D. toledoi*	IBE-DV6	Turkey	Erzurum province, Toprakkaleköyü	TUR3	40°14'22.90"N 40°59'16.70"E	I. Ribera	LN995120	LN995084	LN995190	LN995159			-	
293	*D. toledoi*	IBE-DV7	Turkey	Erzurum province, Toprakkaleköyü	TUR3	40°14'22.90"N 40°59'16.70"E	I. Ribera	**LT602331**	**LT602083**	**LT602609**	**LT602417**	**LT602508**		NO	
294	*D. toledoi*	IBE-DV8	Turkey	Erzurum province, Toprakkaleköyü	TUR3	40°14'22.90"N 40°59'16.70"E	I. Ribera	**LT602332**	**LT602084**					-	
295	*D. toledoi*	IBE-DV116	Turkey	Erzurum province, Toprakkaleköyü	TUR3	40°14'22.90"N 40°59'16.70"E	I. Ribera	**LT602333**	**LT602085**					-	
296	*D. toledoi*	IBE-DV134	Turkey	Erzurum province, Toprakkaleköyü	TUR3	40°14'22.90"N 40°59'16.70"E	I. Ribera	**LT602334**	**LT602086**					-	
297	*D. abnormicollis*	MNCN-AI120	Uzbekistan	Tashkent province, Yakkatut		41º38'N 70º03'E	L. Hendrich	LN995086	LN995059	LN995161	LN995126				
298	*D. algibensis*	NHM-IR76	Spain	Andalucia, Cádiz province, Puerto de Gáliz		ca. 36°33’35”N 5°36’4”W	I. Ribera		AF309318	AF309261					
299	*D. bicostatus*	MNCN-AI639	Portugal	Guarda district, Manteigas (Serra da Estrela)		40º19’57”N 7º37’03”W	I. Ribera	LN995091	HE610179	LN995166	LN995130				
300	*D. depressicollis*	MNCN-AI1023	Spain	Andalucia, Almeria province, Abrucena		ca. 37°8'20"N 2°46'50"W	A. Castro	LN995098	HE610182	LN995172	LN995137				
301	*D. doriae*	MNCN-AI775	Turkey	Bolu province, Kartalkaya		40º39’20”N 31º47’8.5”E	I. Ribera, P. Aguilera & C. Hernando	LN995099	HE610183	LN995173	LN995138				
302	*D. fairmairei*	IBE-DV43	Morocco	Sus-Masa-Draa, Ouarzazate province, Tachokchte		ca. 30°47'36"N 7°31'27"W	I. Ribera & A. Cieslak	LN995101	LN995070	LN995175	LN995140				
303	*D. ferrugineus*	MNCN-AI731	Portugal	Guarda district, Sabugueiro (Serra da Estrela)		40º24’20”N 7º37’43”W	I. Ribera	LN995103	LN995072	LN995176	LN995142				
304	*D. fosteri*	NHM-IR77	Spain	Cataluña, Barcelona province, Saldes		ca. 42°13'36"N 1°44'30"E	P. Aguilera		AF309317	AF309260					
305	*D. hispanicus*	MNCN-AI858	Spain	Comunidad Valenciana, Castellón province, Ballestar		40º41’41”N 0º13’25.5”E	I. Ribera	LN995104	LN995073	LN995177	LN995143				
306	*D. lareynii*	NHM-IR165	France (Corsica)	Haute-Corse department, Vizzavona		ca. 42°6'59"N 9°6'40"E	I. Ribera & A. Cieslak	LN995105	AF309316	AF309259					
307	*D. nilssoni*	IBE-AF104	Iran	Khorasan Shamali province, Eshq Abad		37º 48.2'N 56º55.5'E	J. Hájek & P. Chvojka		LN995080	LN995184	LN995150				
308	*D. opatrinus*	MNCN-AI629	Spain	Andalucia, Córdoba province, Sierra Morena,		38° 5'51.79"N 4°53'34.37"W	A. Castro	LN995112	HE610188	LN995185	LN995151				
309	*D. parvicollis*	MNCN-AI776	Turkey	Bolu province, Kartalkaya		40º39’20”N 31º47’8.5”E	I. Ribera, P. Aguilera & C. Hernando	LN995113	HF931225	HF931454	LN995152				
310	*D. persicus*	NHM-IR45	Iran	Fars province, Sepidan		ca. 30°17'6"N 51°56'54"E	H. Fery		AF309308	AF309251	EF670140				
311	*D. sahlbergi*	MNCN-AI108	Greece (Chios)	Kardamila		ca. 38°31'37"N 26° 5'30"E)	G.N. Foster	LN995117	LN995081	LN995187	LN995156				
312	*D. theryi*	IBE-RA37	Morocco	Taza-Al Hoceima-Taounate,Taza province, Tazzeka National Park		34º08’56.8”N 4º00’25.5”W	I. Ribera, P. Aguilera & C. Hernando	LN995119	LN995083	LN995189	LN995158				
313	*D. wewalkai*	MNCN-AI725	Spain	Castilla-La Mancha, Guadalajara province, Cardoso de la Sierra,		41º05'34.3"N 3º25'32.1"W	I. Ribera & A. Cieslak	LN995121	LN995085	LN995191	LN995160				
314	*D. youngi*	NHM-IR182	Iran	Kohkiluyeh and Boyer Ahmad province, Gachsaran		ca. 30°24'20.78"N 50°50'7.75"	H. Fery		AF309306	AF309249	EF670142				

Table S2.

A) Primers used for the amplification and sequencing. In brackets, length of the amplified fragment.

gene	primer	sequence	ref.
cox1-3’	Jerry (5')	CAACATTTATTTTGATTTTTTGG	(4)
(826)	Pat (3')	TCCAATGCACTAATCTGCCATATTA	(4)
	Chy (5')	T(A/T)GTAGCCCA(T/C)TTTCATTA(T/C)GT	(3)
	Tom (3')	AC(A/G)TAATGAAA(A/G)TGGGCTAC(T/A)A	(3)
barcode	LCO 1490 (5')	GGTCAACAAATCATAAAGATATTGG	(2)
(658)	HCO 2198 (3')	TAAACTTCAGGGTGACCAAAAAATCA	(2)
rrnL+trnL+nad1	16SaR (5')	CGCCTGTTTAACAAAAACAT	(4)
(685-693)	ND1 (3')	GGTCCCTTACGAATTTGAATATATCCT	(4)
H3	H3aF (5')	ATGGCTCGTACCAAGCAGACRCG	(1)
(330)	H3aR (3')	ATATCCTTRGGCATRATRGTGAC	(1)
Wingless	WG550F (5')	ATGCGTCAGGARTGYAARTGYCAYGGYATGTC	(5)
(472-475)	WGAbrZ (3')	CACTTNACYTCRCARCACCARTG	(5)

B) Standard PCR conditions for the amplification of the studied fragments.

step	time	temperature
1	3’	96º
2	30”	94º
3	30”-1’	47-50º *
4	1’	72º
5	Go to step 2 and repeat 34-40 x	
6	10’	72º

* Depending on the annealing temperatures of the primers pair used

References

1. Colgan, D. J., McLauchlan, A., Wilson, G. D. F., Livingston, S. P., Edgecombe, G. D., Macaranas, J., Cassis, G. & Gray, M. R. (1998). Histone H3 and U2 snRNA DNA sequences and arthropod molecular evolution. *Australian Journal of Zoology*, 46: 419-437.
2. Folmer, 0., Black, M., Hoeh, W., Lutz, R. & Vrijenhoek, R. (1994). DNA primers for amplification of mitochondrial cytochrome c oxidase subunit I from diverse metazoan invertebrates. *Molecular Marine Biology and Biotechnology*, 3: 294-299.

3. Ribera, I., Fresneda, J., Bucur, R., Izquierdo, A., Vogler, A.P., Salgado, J.M. & Cieslak, A. (2010). Ancient origin of a Western Mediterranean radiation of subterranean beetles. *BMC Evolutionary Biology*, 10: 29.
4. Simon, C., Frati, F., Beckenbach, A., Crespi, B., Liu, H., & Flook, P. (1994). Evolution, weighting, and phylogenetic utility of mitochondrial gene sequences and a compilation of conserved polymerase chain reaction primers. *Annals of the entomological Society of America*, 87: 651-701.
5. Wild, A. L., & Maddison, D. R. (2008). Evaluating nuclear protein-coding genes for phylogenetic utility in beetles. *Molecular Phylogenetics and Evolution*, 48: 877-891.

Figure S1. Ultrametric time calibrated tree obtained with BEAST with the mitochondrial sequence data of the same specimens used in the analysis of the nuclear sequences (see Fig. S2). Numbers in nodes: Bayesian posterior probabilities. See Table S1 for details on the specimens and localities.

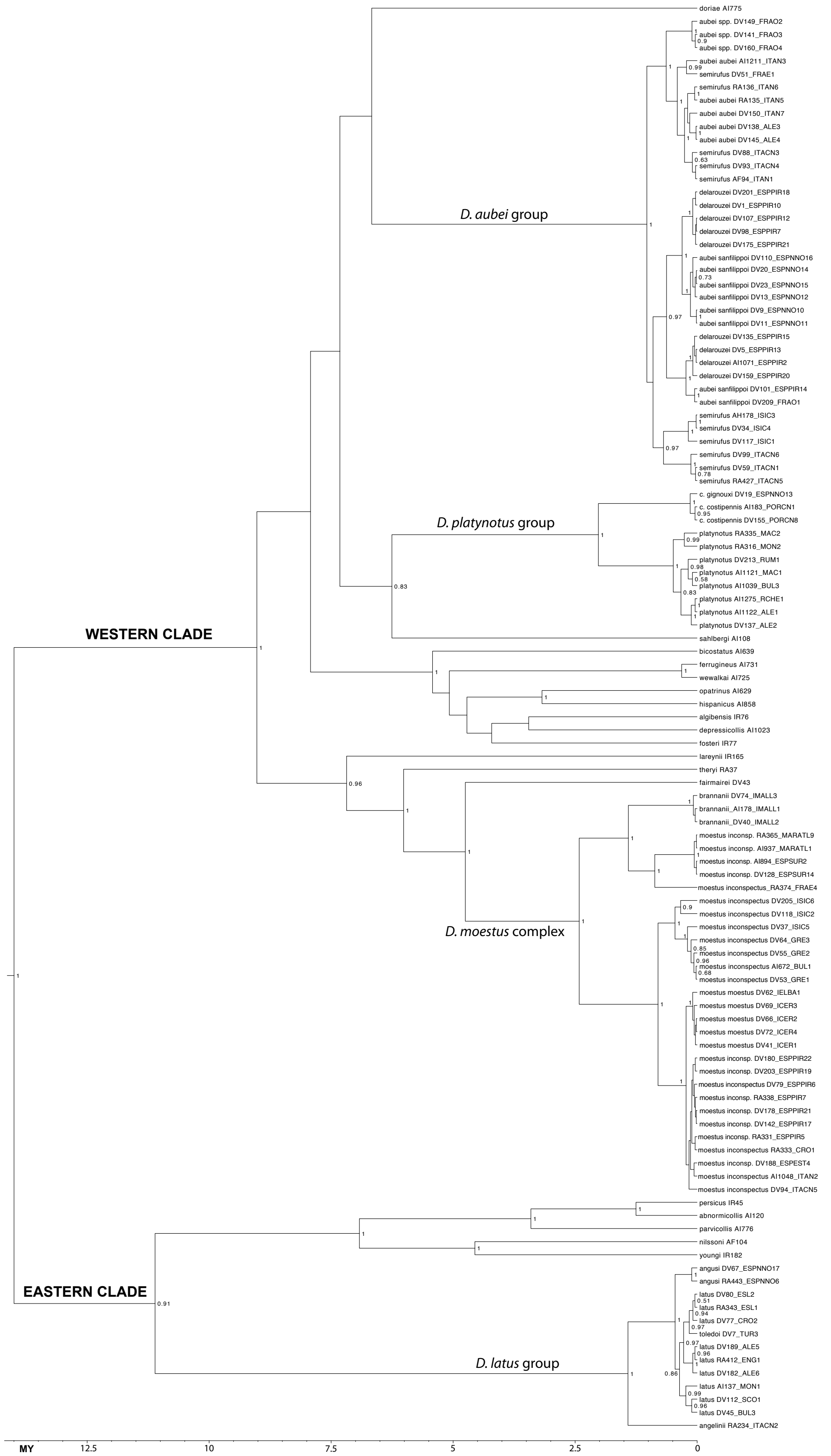

Figure S2. Ultrametric time calibrated tree obtained with BEAST using only the nuclear sequence data of the same specimens used in the analysis of the mitochondrial sequences (see Fig. S1). Numbers in nodes: Bayesian posterior probabilities. See Table S1 for details on the specimens and localities.

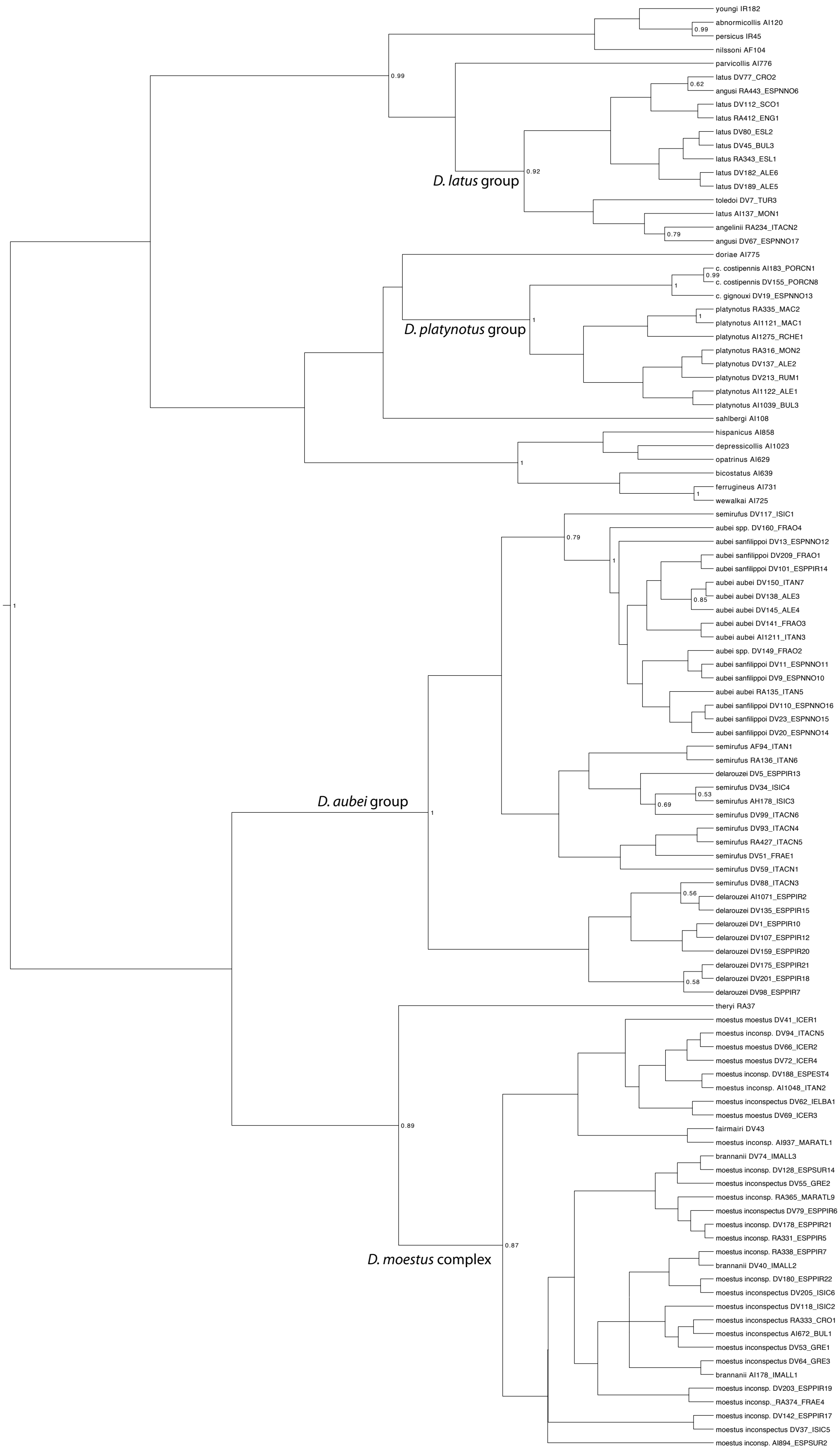

Supplement: Supplemental Information 1 [file peerj-04-2514-s001.pdf]
